# The effects for inflammatory responses by CPP with different colloidal properties in hemodialysis patients

**DOI:** 10.1038/s41598-022-26166-2

**Published:** 2022-12-17

**Authors:** Hideyuki Mukai, Yutaka Miura, Kazuhiko Kotani, Atsushi Kotoda, Hiroshi Kurosu, Toshiyuki Yamada, Makoto Kuro-o, Yoshitaka Iwazu

**Affiliations:** 1 Division of Anti-Aging Medicine, Center for Molecular Medicine, Jichi Medical University, 3311-1 Yakushiji, Shimotsuke, Tochigi 329-0498 Japan; 2 Division of Community and Family Medicine, Center for Community Medicine, Jichi Medical University, Shimotsuke, Tochigi Japan; 3 Seiikai Medical Clinic Oyama, Shimotsuke, Tochigi Japan; 4 Department of Clinical Laboratory Medicine, Jichi Medical University, Shimotsuke, Tochigi Japan

**Keywords:** Biomarkers, Diagnostic markers, Predictive markers, Prognostic markers, Cardiovascular biology, Calcification, Pathogenesis, Inflammation, Chronic inflammation, Nephrology, Renal replacement therapy, Haemodialysis

## Abstract

Calciprotein particles (CPPs) are colloids composed of solid-phase calcium-phosphate and serum protein fetuin-A. CPPs form a polydispersed system with different particle size and density. CPPs with specific physical properties can induce calcification and innate immune responses in cultured cells. In hemodialysis patients, blood CPP levels were reported to correlate with vascular calcification and inflammation. However, little is known about relation between these disorders and physical properties of CPPs. Here, we show that the association between physical properties of plasma CPPs and serum levels of inflammatory cytokines/chemokines in 78 hemodialysis out-patients by cross-sectional study. Patients with cardiovascular disease (CVD) had significantly higher high density CPP (H-CPP) levels than patients without CVD but not low density CPP (L-CPP). Seven cytokines/chemokines (EGF, eotaxin, IL-8, IP-10, MCP-1, MIP-1, MIP-1β and TNFα) were detectable in the serum samples from > 95% of the patients. In multivariate regression analysis, H-CPP was positively associated with eotaxin after adjusting for age, gender, smoking, serum phosphate and FGF23. L-CPP was negatively associated with IL-8 after adjusting for age, gender, serum albumin, phosphate and FGF23. High H-CPP levels were associated with pro-inflammatory response, whereas L-CPPs were associated with anti-inflammatory response. CPPs with different physical properties may impact differently on pathophysiology in HD patients.

## Introduction

Cardiovascular disease (CVD) is the leading cause of death and highly prevalent in patients with chronic kidney disease (CKD)^1–3^. In addition to traditional risk factors for CVD, several non-traditional CKD-specific risk factors have been identified, including mineral bone disorders (MBD) and inflammation^4–6^. CKD- MBD increases the risk for ectopic calcification, particularly vascular calcification (VC)/ arterial calcification (AC)^7^. VC/AC in general population as well as in CKD patients is predictive of subsequent CVD morbidity and mortality beyond established conventional risk factors^8–10^. Hyperphosphatemia has been identified as an independent risk for VC/AC. However, the mechanism by which hyperphosphatemia leads to VC/AC remains elusive^10–13^.

Recent studies have identified calciprotein particles (CPPs) as a potential mediator between hyperphosphatemia and VC/AC. CPPs are mineral-protein complexes mainly composed of serum protein fetuin-A laden with solid-phase calcium-phosphate (CaPi) and dispersed in the blood as colloids. Clinical studies demonstrated that circulating CPP levels correlated positively with serum phosphate levels and parameters for AC/VC (coronary artery calcification scores and aortic pulse wave velocity) in CKD patients and cardiovascular events in hemodialysis (HD) patients^14^. Furthermore, in vitro studies showed that synthesized CPPs induced calcification in cultured vascular smooth muscle cells (VSMCs)^15^. These observations have raised the possibility that CPPs may be a causative agent of VC/AC^16^.

The process of CPP formation and maturation has been extensively studied in vitro. An increase in concentration of calcium and phosphate beyond the solubility limit leads to precipitation of amorphous CaPi. In the presence of serum, the amorphous CaPi precipitates are adsorbed by serum protein fetuin-A and prevented from further growth. Consequently, fetuin-A molecules bound to tiny amorphous CaPi precipitates are generated, which are termed primary CPPs. Amorphous CaPi precipitates in primary CPPs spontaneously undergo amorphous-to-crystalline phase transition to increase the density of CPPs over time. The CPPs containing crystalline CaPi are designated as secondary CPPs. Because the phase transition is coupled with self-aggregation of CPPs^17^, secondary CPPs are larger in particle size and higher in density than primary CPPs^18–21^. CPPs have been classified by two different axes that are crystallinity and density (Supplementary Fig. 1). Primary CPPs and secondary CPPs are classified based on their crystallinity. On the other hand, low density CPP (L-CPP) and high density CPP (H-CPP) are classified based on their density. Namely, H-CPPs are defined as CPPs that can be precipitated by centrifugation at 16,000 g for 2 h, whereas L-CPPs are defined as CPPs that cannot be precipitated by centrifugation at 16,000 g for 2 h. The gel-filtration assay uses a probe that binds not to amorphous CaPi but to crystalline CaPi^18^. Therefore, both H-CPPs and L-CPPs detected by the gel-filtration method are regarded as secondary CPPs by definition.

Recent in vitro studies indicated that secondary CPPs had the ability to induce inflammatory responses in cultured macrophages^22–24^. Enhanced macropinocytosis of CPPs with larger particle size resulted in increased lysosomal activity, nucleotide-binding oligomerization domain, leucine-rich repeat and pyrin domain containing 3 (NLRP3) inflammasome activation, and interleukin (IL) -1β release^25^. Secondary CPPs induced the expression and release of tumor necrosis factor (TNF) α, which enhanced calcification via its receptor TNFR1^15^. Thus, CPPs with large particle size and high density functioned as a potent inducer of inflammation in vitro. However, it remains to be determined which colloidal properties of CPPs may be associated with chronic inflammation.

CKDis associated with high CVD prevalence, high circulating CPP levels, and chronic inflammation^26^. In the present study, we tested whether CPPs with different colloidal properties might be associated with different clinical manifestations in maintenance HD patients.

## Materials and methods

### Patients and study design

Circulating levels of CPPs, cytokines and chemokines were measured in 78 clinically stable HD out-patients recruited from a clinic (Seiikai medical clinic Oyama, Tochigi, Japan) between October 2016 and November 2016. Exclusion criteria were age < 18 years, signs of overt clinical infection and unwillingness to participate. All patients were treated with the use of high-flux membranes^27^, and the median blood flow was 230 (10–90th percentile 200–300) ml/min, the median duration of each dialysis session was 4.5 (10–90th percentile 3.8–5.5) hours, and the calcium level of the dialysate of all patients was 3.0%. Ultrapure dialysis fluid was managed in according to the standards of Japanese Society for Dialysis Therapy. Written Informed consent was obtained from each patient. The Ethics Committee in Jichi Medical University approved study protocols. The studies were conducted in adherence with the Declaration of Helsinki.

### Quantification of plasma calciprotein particles (CPPs)

Circulating CPP levels have been quantified by the “fetuin-A method”: Serum/plasma samples are centrifuged at 16,000 g for 2 h to precipitate CPPs. The difference in the serum/plasma fetuin-A levels determined by ELISA between before and after the centrifugation is assumed to represent the CPP level. Thus, the fetuin-A method measures CPPs over a certain density that can be precipitated by the centrifugation at 16,000 g for 2 h. On the other hand, we recently developed a novel CPP assay termed “gel-filtration method”^18^ and found that decent amount of secondary CPPs were present in the supernatant of serum/plasma samples centrifuged at 16,000 g for 2 h. These CPPs were designated as L-CPPs. The difference in the fluorescence intensity before and after the centrifugation correlated with the CPP level determined by the fetuin-A method and designated as H-CPPs.

In the present study, CPPs in blood samples were quantified by the gel-filtration method. Preparation of plasma samples was strictly standardized to avoid potential variation of the amorphous-to-crystalline phase transition of CaPi in CPPs in vitro. Blood was drawn immediately before dialysis at two-days of interval using heparinized blood collecting tubes. The blood samples were centrifuged at 3000 rpm for 10 min to separate plasma within 60 min after sampling. The plasma samples were aliquoted in microcentrifuge tubes, snap-frozen in liquid nitrogen, and stored at − 80 °C. The frozen plasma samples were thawed 24 h before starting the CPP assay and incubated at 25 °C to convert amorphous CaPi in CPPs to crystalline CaPi. After incubation for 22 h, each sample was divided into two tubes. One of the tubes was centrifuged at 16,000 g for 2 h at 25 °C. The supernatant was used for measurement of theL-CPPlevel. The other tube was left untreated at 25 °C for 2 h and then used for measurement of the total CPP (T-CPP) level.

A fluorescent probe that binds to CaPi crystals (OsteoSense 680EX; PerkinElmer Inc., Waltham, MA) was added to plasma samples. After incubation at 25 °C for 60 min, the sample was applied to a gel-filtration spin column to remove unbound OsteoSense. The amount of CPPs in the sample was expressed as the fluorescence intensity of the flow-through fraction quantified using an infrared fluorescence scanner (Odyssey CLx; LICOR Biosciences, Lincoln, NE). The H-CPP level was calculated by subtracting the L-CPP level from the T-CPP level. In case that calculated H-CPP was less than zero, the H-CPP level was designated as zero.

### Measurements of serum cytokine and chemokine levels

Serum cytokine and chemokine levels were measured using suspension array technology in multiplex using a MILLIPLEX® map 29 premix kit for human eotaxin (eotaxin-1, CCL11), granulocyte colony stimulating factor (G-CSF), granulocyte–macrophage colony stimulating factor (GM-CSF), interferons (IFN)-α2, IFN-γ, Interleukin (IL)-10, IL-12 (p40), IL-12 (p70), IL-13, IL-15, IL-17, IL-1α, IL-1β, IL-2, IL-3, IL-4, IL-5, IL-6, IL-7, IL-8, interferon gamma-induced protein (IP)-10, monocyte chemotactic protein (MCP)-1, macrophage inflammatory protein (MIP)-1α, MIP-1β, tumor necrosis factor (TNF)-α and TNF-β (Millipore Corp, St Charles, Missouri, USA) according to the manufacturer’s instructions.

### Blood sampling and laboratory analysis

Serum levels of albumin, triglycerides, total cholesterol, high-density lipoprotein (HDL)-cholesterol, calcium, phosphate, C-reactive protein, plasma intact-parathyroid hormone (PTH) and hemoglobin were analyzed using certified methods at the clinic. Low-density lipoprotein (LDL)-cholesterol was calculated using the Friedewald formula: [(total cholesterol)—(HDL-cholesterol)—(triglycerides/5)]^28^. Serum intact fibroblast growth factor 23 (FGF23) levels were measured using an enzyme-linked immunosorbent assay kit (Kainos Laboratories, Inc., Tokyo, Japan).

### Clinical assessments

Presence of CVD was defined as history or signs of ischemic heart disease, peripheral vascular disease, and/or cerebrovascular disease. Body mass index (BMI) was calculated as weight in kilograms divided by the square of height in meters.

### Statistical analyses

Data are expressed as median (10th to 90th percentile) or percentage. Statistical significance was set at the level of *p* < 0.05. Comparisons between two groups were performed by non-parametric Wilcoxon test for continuous variables and Chi-square test for nominal variables. Non-parametric Spearman rank correlation analysis was used to determine associations between variables. Multiple linear regression analyses were performed for continuous variables of H-CPPs and L-CPPs. The results were shown as standardized β regression coefficients. Parameters showing significant association in bivariate analyses were used for adjustment in multiple linear regression analyses. Statistical analyses were performed using statistical software JMP 14 (SAS institute Inc., Cary, NC, USA) and Stata 16.0 (Stata Corporation, College Station, TX, USA).

## Results

### Clinical and laboratory characteristics

Clinical and biochemical characteristics of 78 HD out-patients are presented in Table 1. Their ages ranged from 26 to 78 years. The median age of the patients was 62 years, 67% were males. 41% had diabetes mellitus (DM). The etiologies of renal disease were chronic glomerulonephritis (*n* = 22; 28%), hypertension and renovascular disease (*n* = 6; 8%), polycystic kidney disease (*n* = 9; 11%), diabetic nephropathy (*n* = 28; 36%) and others or unknown causes (*n* = 13; 17%). The median dialysis vintage was 59 months. Clinical signs or symptoms of CVD were present in 18% of the patients (cerebrovascular disease: 5 patients, ischemic heart disease: 5 patients, and peripheral artery disease: 6 patients). The median plasma T-CPP, L-CPP and H-CPP levels were 279,006, 149,838 and 124,418 AU, respectively.

**Table 1:** Baseline clinical and biochemical characteristics of 78 HD patients.

**Demography and clinical characteristics**	
Age (years)	62 (45–70)
Males, n (%)	52 (67)
Dialysis vintage (month)	59 (14–246)
Diabetes mellitus, n (%)	32 (41)
Cardiovascular disease^a^, n (%)	14 (18)
Current smoker, n (%)	12 (15)
Systolic blood pressure (mmHg)	142 (113–165)
Body mass index, (kg/m^2^)	22.7 (17.9–30.3)
**Biochemicals**	
Hemoglobin (g/dL)	11.3 (9.9–12.9)
Albumin (g/dL)	3.5 (3.1–4.0)
Triglyceride (mg/dL)	108 (60–187)
Total cholesterol (mg/dL)	171 (131–226)
HDL cholesterol (mg/dL)	53 (35–75)
LDL cholesterol (mg/L)	102 (59–137)
Calcium (mg/dL)	8.9 (8.1–9.8)
Phosphate (mg/dL)	5.7 (3.9–7.0)
Intact-PTH (ng/L)	126 (40–245)
FGF23 (pg/mL)	3699 (373–19.482)
C-reactive protein (mg/dL)	0.06 (0.01–0.38)
β2- microglobulin (mg/L)	25.9 (18.3–35.4)
**Medication**s	
Statins, n (%)	14 (18)
ACEi/ARB, n (%)	44 (56)
β-blockers, n (%)	26 (33)
Ca-blocker, n (%)	32 (41)
Diuretics, n (%)	41 (53)
Calcium carbonate, n (%)	48 (62)
Sevelamer hydrochloride, n (%)	25 (32)
Ferric citrate hydrate, n (%)	22 (28)
Sucroferric oxyhydroxide, n (%)	22 (28)
Cinacalcet, n (%)	23 (29)
**Calciprotin particles**	
Total calciprotein particle (AU)	279,006 (75,506–340,808)
Low density calciprotein particle (AU)	149,838 (70,608–197,568)
High density calciprotein particle (AU)	124,418 (1372–182,194)

Continuous variables are presented as median (10–90 percentile). Categorical variables are presented as number (n)/percentage (%). ^a^Presence of cardiovascular disease was defined as clinical history or signs of ischemic cardiac disease, and/or presence of peripheral vascular disease and/or cerebrovascular disease. *HDL* high-density lipoprotein; *LDL* low-density lipoprotein, *calculated based on Friedewald formula* (total cholesterol)—(high-density lipoprotein cholesterol—(triglycerides/5); *intact-PTH* intact parathyroid hormone; *FGF23* Fibroblast growth factor-23; *ACEi* angiotensin-converting enzyme inhibitor; *ARB* angiotensin-2 receptor blocker.

### Association of CPPs with the prevalence of CVD

**Figure 1:**
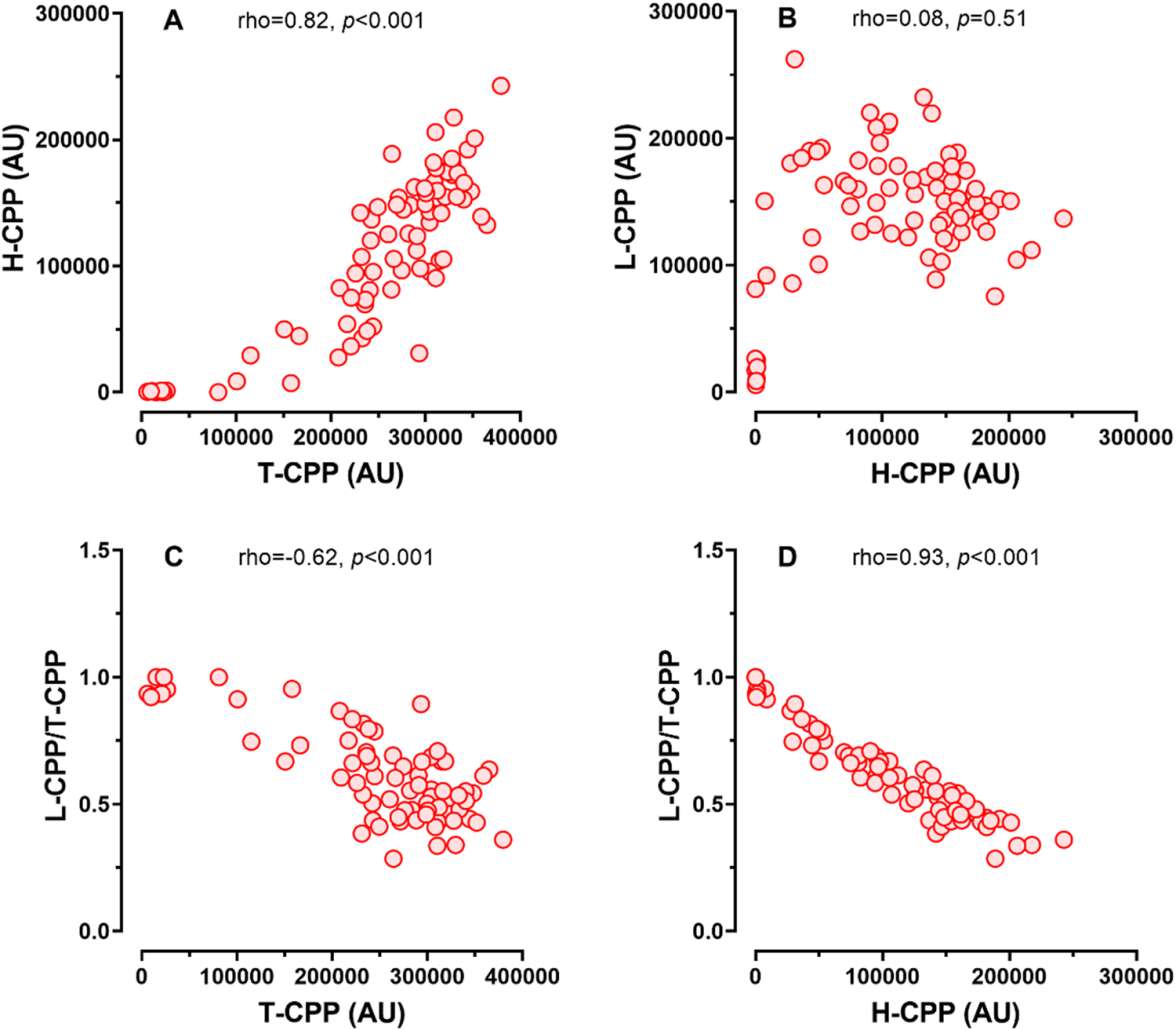
Correlations between H-CPP, T-CPP, L-CPP, and L-CPP/T-CPP among 78 HD patients. Plasma H-CPP levels were positively correlated with T-CPP levels (**A**), but not with L-CPP levels (**B**), the ratio of L-CPP to T-CPP was significantly decreased as the T-CPP (**C**) or H-CPP levels (**D**) were increased. Rho and *p* values were indicated.

**Figure 2:**
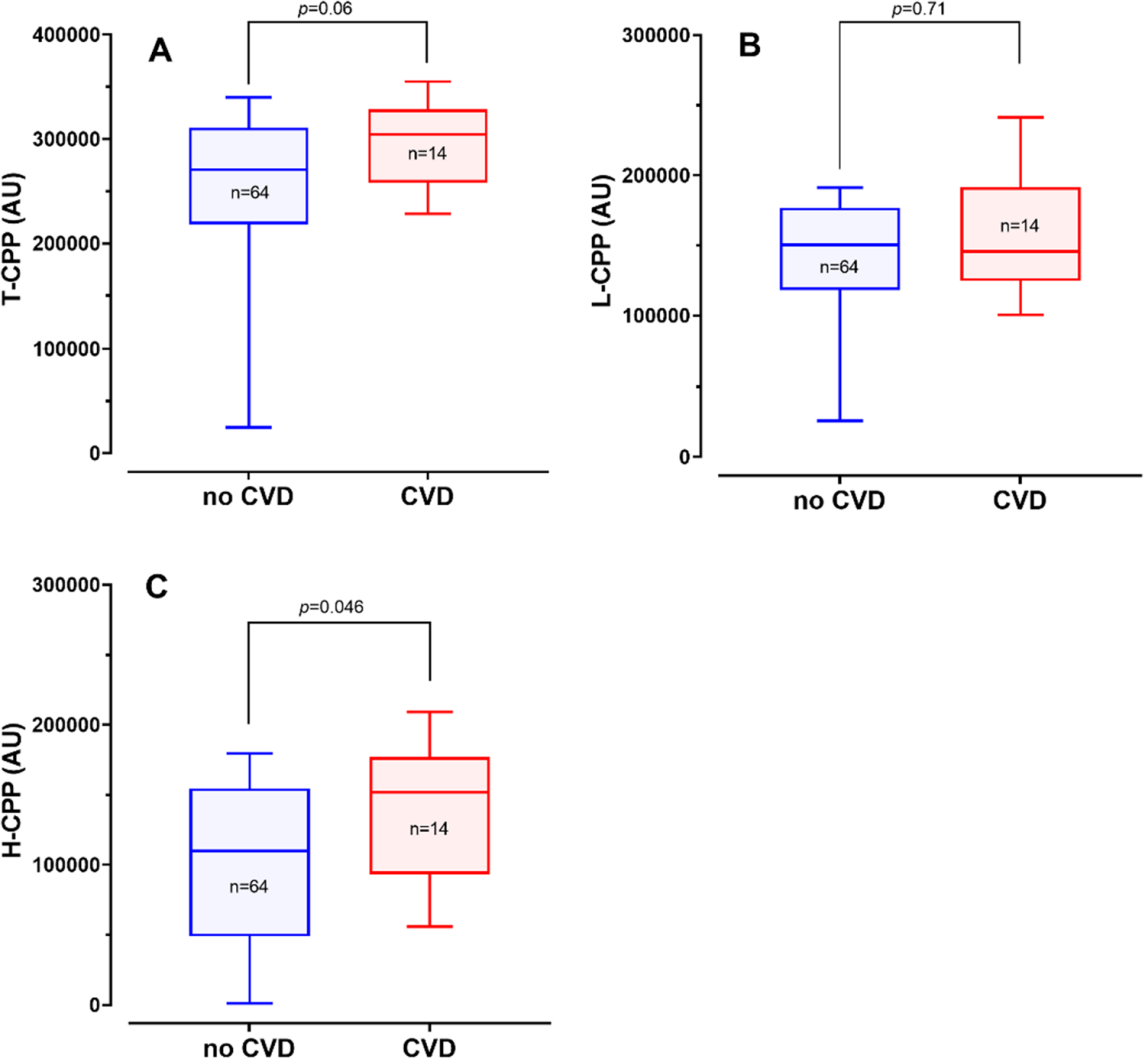
Comparison of CPP levels between patients with and without cardiovascular disease (CVD)**.** Box plots showing plasma levels of T-CPP (**A**), L-CPP (**B**), and H-CPP (**C**) in patients with CVD (CVD) and without CVD (no CVD). The number of patients (n) and *p* values by Wilcoxon test are indicated.

Plasma H-CPP levels were positively correlated with T-CPP levels, but not with L-CPP levels (Fig. 1A, B), indicating that the increase in T-CPP levels could be explained primarily by the increase in H-CPP levels, but not L-CPP levels. Accordingly, the ratio of L-CPP to T-CPP was significantly decreased as the T-CPP or H-CPP levels were increased (Fig. 1C, D). Patients with CVD had significantly higher H-CPP levels and tended to have higher T-CPP levels than patients without CVD. However, L-CPP levels were not different between patients with or without CVD (Fig. 2).

### Correlations of CPPs with serum cytokine/chemokine levels

Seven (7) out of 29 cytokines/chemokines (EGF, eotaxin, IL-8, IP-10, MCP-1, MIP-1β, and TNFα) were detectable in more than 95% of the patients (Supplementary Table 1). Correlations between serum levels of these cytokines/chemokines and plasma levels of CPPs in 78 HD patients was presented in Table 2. In bivariate analysis, serum eotaxin levels were correlated positively with plasma H-CPP levels (rho = 0.31, *p* = 0.005). Serum IL-8 levels were correlated negatively with plasma L-CPP levels (rho = −0.28, *p* = 0.01). The other detectable chemokines did not show any significant correlation with CPPs.

**Table 2:** bivariate correlations expressed as rho correlations of EGF, Eotaxin, IL-8, IP-10, MCP-1, MIP-1β and TNFα with CPPs at baseline in 78 HD patients.

	T-CPP		L-CPP		H-CPP	
	rho	*p* value	rho	*p* value	rho	*p* value
EGF	0.02	0.87	− 0.08	0.50	0.06	0.62
Eotaxin	0.21	0.06	0.16	0.16	**0.31**	**0.005**
IL-8	− 0.15	0.18	− **0.28**	**0.01**	− 0.03	0.79
IP-10	− 0.12	0.30	− 0.17	0.14	− 0.04	0.72
MCP-1	0.08	0.48	− 0.16	0.17	0.18	0.12
MIP-1β	0.04	0.75	0.005	0.97	− 0.07	0.58
TNFα	− 0.04	0.75	− 0.10	0.37	− 0.01	0.91

Signigficant values are in bold. *T-CPP* total calciprotein particle; *L-CPP* low density calciprotein particle; *H-CPP* High density calciprotein particle; *EGF* epidermal growth factor; *IL-* interleukin-; *IP-10* Interferon gamma-induced protein 10 (C-X-C motif chemokine ligand 10); *MCP-1* Monocyte chemoattractant protein − 1; *MIP-1α* macrophage inflammatory protein 1α; *MIP-1β* Macrophage inflammatory protein-1β; *TNFα* tumor necrosis factor α.

### Bivariate correlations of eotaxin and IL-8 with clinical parameters

In bivariate analysis, serum eotaxin levels positively correlated with HD vintage (rho = 0.22, *p* = 0.046) and serum FGF23 levels (rho = 0.23, *p* = 0.046). Serum IL-8 levels were negatively correlated with serum albumin (rho = −0.22, *p* = 0.048) and β2-microglobulin levels (rho = 0.35, *p* = 0.002) (Supplementary Table 2).

### Bivariate correlations of H-CPP and L-CPP with clinical parameters

In bivariate analysis, plasma H-CPP levels were significantly correlated with the presence of CVD (rho = 0.23, *p* = 0.046), current smoking (rho = 0.29, *p* = 0.01), serum phosphate (rho = 0.39, *p* < 0.001), FGF23 (rho = 0.41, *p* < 0.001), and β2-microglobulin (rho = 0.25, *p* = 0.03). The plasma L-CPP levels were significantly correlated with dialysis vintage (rho = -0.24, *p* = 0.04), BMI (rho = 0.30, *p* = 0.007), serum levels of albumin (rho = 0.23, *p* = 0.04), triglyceride (rho = 0.34, *p* = 0.002), calcium (rho = 0.25, *p* = 0.03), phosphate (rho = 0.27, *p* = 0.02), FGF23 (rho = 0.25, *p* = 0.03), and β2-microglobulin (rho = -0.25, *p* = 0.03) (Supplementary Table 3and Supplementary Fig. 2).

### Multivariate analysis

Multivariate regression models were used to determine independent significant predictors of H-CPP and L-CPP (Table 3A, B). The positive association between serum eotaxin levels and plasma H-CPP levels remained significant after multiple adjustments including age, gender, smoking, serum phosphate and FGF23. The negative association between serum IL-8 levels and plasma L-CPP levels remained significant after multiple adjustments including age, gender, smoking, serum levels of albumin, phosphate and serum FGF23.

**Table 3:** Multiple regression models for 1-SD increase of H-CPP (**A**) and 1-SD increase of L-CPP (**B**) in 78 HD patients.

**A**						
	Model 1			Model 2		
	Age, sex, smoking adjusted (Adjusted *r*^2^ = 0.11, *p* = 0.01)			Multiple adjusted (Adjusted *r*^2^ = 0.22, *p* < 0.001)		
	β	*p*	95%CI	β	*p*	95%CI
1-SD higher eotaxin	**0.27**	**0.01**	0.05–0.49	**0.23**	**0.03**	0.03–0.44
1-SD higher age	− 0.01	0.92	− 0.23–0.20	0.03	0.79	− 0.18–0.23
Male vs female	0.04	0.70	− 0.38–0.57	0.003	0.98	− 0.45–0.46
Current smoking (yes/no)	**0.24**	**0.03**	0.05–1.29	0.20	0.07	− 0.04–1.16
1-SD higher phosphate	–			**0.30**	**0.01**	0.07–0.53
1-SD higher FGF23	–			0.12	0.28	− 0.11–0.37

**B**						
	Model 1			Model 2		
	Age, sex, albumin adjusted (Adjusted *r*^2^ = 0.04, *p* = 0.16)			Multiple adjusted (Adjusted *r*^2^ = 0.11, *p* = 0.02)		
	β	*p*	95%CI	β	*p*	95%CI
1-SD higher IL-8	− **0.23**	**0.047**	− 0.61– − 0.004	− **0.23**	**0.04**	− 0.61– − 0.02
1-SD higher age	0.06	0.60	− 0.17–0.29	0.08	0.46	− 0.14–0.31
Male vs female	0.01	0.92	− 0.47–0.52	− 0.02	0.85	− 0.53–0.44
1-SD higher albumin	0.17	0.17	− 0.07–0.41	0.12	0.33	− 0.12–0.36
1-SD higher phosphate	–			0.21	0.09	− 0.04–0.45
1-SD higher FGF23	–			0.17	0.16	− 0.61– − 0.02

Signigficant values are in bold. *β* standardized regression coefficient; 95%CI, 95% confidence interval for regression coefficient; *SD* standard deviation.

## Discussion

The present study demonstrated that CPPs with different colloidal properties were correlated with different clinical parameters and inflammatory responses. First, plasma H-CPP levels associated positively with serum levels of eotaxin. Second, plasma L-CPP levels associated negatively with serum IL-8 levels after adjusting with possible co-variates.

Gatate et al. demonstrated that plasma T-CPP levels were associated with CVD events in hemodialysis patients under a prospective design^14^. Because T-CPP levels were positively correlated with plasma H-CPP levels (Fig. 1), we expected to observe that patients with CVD should have higher H-CPP and T-CPP levels than patients without CVD. As expected, we observed that the patients with CVD had higher H-CPP levels than the patients without CVD (Fig. 2). However, the difference in the T-CPP levels did not reach statistical significance (*p* = 0.06). This may be due to the small number of patients enrolled in the present study and the different (prospective vs cross-sectional) study design. Further studies are required to determine the association of CPPs with different physical property with clinical outcomes.

The eosinophil chemokine eotaxin (also known as CCL11) is highly expressed in human atherosclerotic plaques^29^. An increase in serum eotaxin levels were reportedly associated with coronary artery disease, suggesting that eotaxin may contribute to vascular inflammation^29–31^. In addition, we found negative correlation between L-CPPs and serum levels of IL-8, which is known to participate in pathogenesis of CVD^32^. Prospective clinical studies demonstrated that an increase in plasma IL-8 concentration was associated with coronary artery calcifications, identifying IL-8 as a powerful prognostic predictor of all-cause and cardiovascular mortality in CKD patients^33,34^. In the present study, serum IL-8 levels were correlated positively with serum β2-microglobulin levels and negatively with serum albumin levels (Supplementary Table 2). IL-8 may be one of the inflammatory mediators linking between uremic toxin, malnutrition, and vascular calcification in CKD. Recently, Perna et al. reported that increased plasma levels of eotaxin and IL-8 were associated with low GFR and vascular calcification^35^. These findings indicate that H-CPPs and L-CPPs may exert opposite effects on inflammation. Namely, it is intriguing to speculate that H-CPPs and L-CPPs may behave like pro-inflammatory and anti-inflammatory factors, respectively.

Eotaxin expression is upregulated in tissues with allergic inflammation and associated with eosinophil infiltration and disease severity^36,37^. Eotaxin mRNA levels were reported to be increased not only in the skin of atopic dermatitis patients but also in biopsies from itchy skin lesions when eosinophils are present ^38^. The immune system dysregulation and inflammation are one of the possible mechanisms for generation of pruritis^39^. Chronic kidney disease-associated pruritus (CKD-aP) is a common symptom and impairs Health-Related Quality of Life (HRQoL) and clinical outcomes in patients undergoing dialysis^40–43^. Increased eosinophils have also been observed in non-dialysis patients with CKD-aP^44^. Keithi-Reddy et al. reported a case of uremic pruritus who had eosinophilia and eosinophil infiltration in the skin biopsy^45^. The pathophysiology of CKD-aP is complex and incompletely understood. A hypothesis on the mechanism of CKD-aP implicated toxins which include calcium, phosphate and magnesium in the skin and subcutaneous tissue as potential pruritogens. This hypothesis was based on several observations,e.g., the association of CKD-aP with higher calcium and phosphate levels,and the improvements in pruritus after treatment of high calcium and phosphate levels. However, subsequent studies have not confirmed these associations^46^. Thus, no information on the possible involvement of H-CPPs and eotaxin in CKD-aP is available so far. Further studies are required to clarify the roles that CPPs, eotaxin and eosinophils may play in pathogenesis of CKD-aP.

Limitations of the present study include the relatively low number of participants and its observational cross-sectional design that does not permit conclusions concerning causality. Second, we did not investigate several other potential determinants of eotaxin level, such as immunoglobulin E (IgE), eosinophil count, and symptoms of pruritus, which are common in HD patients. Third, we explored only cytokines/chemokines which were detected by using Milliplex® map 29 premix kit. Using other measuring method could have identified other cytokines/chemokines associated with CPPs. However, this method has the advantage of assessing a large number of immune modulators simultaneously, which enabled us to observe different aspects of immune response. Fourth, in this study, we did not perform the T_50_ test, which reflects propensity for transformation from primary CPPs to secondary CPPs^47^.

Moreover, as we reported previously, circulating CPPs in vivo barely contain crystalline CaPi ^18^, probably because CPPs containing crystalline CPPs are phagocytosed by Kupffer cells and macrophages to be removed from circulation^16^. However, the gel-filtration assay can measure only CPPs containing crystalline CaPi. Therefore, to quantify serum CPP levels in clinical samples by the gel-filtration assay, we must transform the amorphous CaPi to crystalline CaPi in vitro prior to the assay. We identified several procedures that promoted the amorphous-to-crystalline phase transition of CaPi in CPPs, including blood coagulation, incubation at room temperature, and freeze-thaw^18^. The amorphous CaPi should have been transformed to crystalline CaPi during the sample processing in vitro, which enabled us to quantify serum CPP levels by the gel-filtration assay. We also reported that the amorphous-to-crystalline phase transition was accompanied by aggregation of the CPPs, which increased the particle size and density^18^. Thus, the sample processing standardized in this study not only induced the amorphous-to-crystalline phase transition but also increased the density of CPPs, thereby facilitating formation of H-CPPs. H-CPPs detected by the gel-filtration assay may be viewed as artifacts generated in vitro during the sample processing. However, the H-CPP levels potentially reflect the nature of circulating CPPs in vivo. Namely, high H-CPP levels may indicate that the circulating CPPs in vivo was abundant and highly aggregated, which might have contributed to inflammatory responses.

In summary, the present study demonstrated that serum eotaxin levels were positively associated with the plasma H-CPP levels and that serum IL-8 levels were negatively associated with the plasma L-CPP levels in HD patients. These findings suggest that inflammatory responses induced by CPPs may depend on their colloidal properties. Further studies on mechanisms behind these associations in CKD are warranted.

## Supplementary Information

Supplementary Information.

## Data Availability

On reasonable request, derived data supporting the findings of this study are available from the corresponding author after approval from the Ethical Committee of the Jichi Medical University.
