# Supplementary material for: The effects for inflammatory responses by CPP with different colloidal properties in hemodialysis patients
Source: Sci Rep. 2022 Dec 17;12:21856. doi: 10.1038/s41598-022-26166-2 (PMC9759584; doi:10.1038/s41598-022-26166-2)
Supplement: Supplementary file 1 — Supplementary Information. [file 41598_2022_26166_MOESM1_ESM.pdf]

The effects for inflammatory responses by CPP with different colloidal properties in hemodialysis patients

Hideyuki Mukai[1], Yutaka Miura[1], Kazuhiko Kotani[2], Atsushi Kotoda[3], Hiroshi Kurosu[1], Toshiyuki Yamada[4], Makoto Kuro-o[1], Yoshitaka Iwazu[1*]

[1] Division of Anti-Aging Medicine, Center for Molecular Medicine, Jichi Medical University, Shimotsuke, Tochigi, Japan

[2] Division of Community and Family Medicine, Center for Community Medicine, Jichi Medical University, Shimotsuke, Tochigi, Japan

[3] Seiikai medical Clinic Oyama, Shimotsuke, Tochigi, Japan

[4] Department of Clinical Laboratory Medicine, Jichi Medical University, Shimotsuke, Tochigi, Japan

Corresponding author: Yoshitaka Iwazu, M.D. PhD.

Address: 3311-1 Yakushiji, Shimotsuke, 329-0498 Tochigi, Japan.

Telephone: +81-285-58-7449, Fax: +81-285-44-7322

Email: iwazu@jichi.ac.jp

Supplementary Table 1. Serum cytokines and chemokines profiles of 78 HD patients.

Chemokines	Detectable (n, %)	Value[a] (pg/ml)
EGF	77 (99)	190 (76-461)
Eotaxin	78 (100)	173 (73-305)
G-CSF	28 (36)	12.3 (6.2-246.7)
GM-CSF	15 (19)	8.2 (3.8-96.1)
IFNα2	14 (18)	19.1 (5.2-54.3)
IFN-γ	25 (32)	12.2 (4.2-116.9)
IL-10	27 (35)	5.0 (3.4-15.4)
IL-12p40	30 (38)	14.1 (4.5-46.5)
IL-12p70	11 (14)	21.5 (4.5-87.4)
IL-13	5 (6)	25.8 (11.9-74.2)
IL-15	8 (10)	5.2 (3.3-11.6)
IL-17	21 (27)	14.1 (5.1-64.5)
IL-1ra	53 (68)	12.1 (4.9-53.3)
IL-1a	14 (18)	11.0 (5.4-147.5)
IL-1b	5 (6)	5.5 (3.2-10.6)
IL-2	4 (5)	13.9 (6.9-163.6)
IL-3	0 (0)	-
IL-4	5 (6)	56.0 (6.4-143.8)
IL-5	4 (5)	12.5 (5.0-23.5)
IL-6	23 (29)	6.2 (2.5-36.6)
IL-7	8 (10)	7.4 (3.5-70.8)
IL-8	78 (100)	14.6 (7.2-34.0)
IP-10	78 (100)	395 (150-923)
MCP-1	78 (100)	693 (214-1475)
MIP-1α	39 (50)	6.6 (3.3-20.4)
MIP-1β	74 (95)	34.2 (18.4-59.1)
TNFα	78 (100)	36.9 (22.8-56.9)
TNFβ	7 (9)	6.7 (4.2-27.5)
VEGF	57 (73)	92 (18-464)

Continuous variables are presented as median (10 – 90 percentile). Categorical variables are presented as number (n)/percentage (%). [a] calculated as a median among the detectable levels. Abbreviations: H-CPP, High

density Calciprotein particle; EGF, epidermal growth factor; G-CSF, granulocyte-colony stimulating factor; GM-CSF, granulocyte-macrophage colony-stimulating factor; IFNα2, Interferon α2; IL-, interleukin- ; IP-10, Interferon gamma-induced protein 10 (C-X-C motif chemokine ligand 10); MCP-1, Monocyte chemoattractant protein−1; MIP-1α, macrophage inflammatory protein 1α; MIP-1β, Macrophage inflammatory protein-1β; TNFα, tumor necrosis factor α; TNFβ, tumor necrosis factor β; VEGF, vascular endothelial growth factor.

Supplementary Table 2. Bivariate correlations expressed as rho correlations of Eotaxin and IL-8 with other variables at baseline in 78 HD patients.

	Rho correlations	
	Eotaxin	IL-8
Age (years)	0.10	0.04
Males (%)	0.09	-0.11
Dialysis vintage (month)	**0.22**[a]	0.20
Diabetes mellitus (present)	0.05	-0.17
Cardiovascular disease (present)	0.12	-0.10
Current Smoking	0.06	0.03
Systolic blood pressure (mmHg)	0.06	-0.17
Body mass index (kg/m^2)	-0.19	-0.20
Hemoglobin (g/dL)	0.07	0.20
Albumin (g/dL)	-0.04	**-0.22**[a]
Total Cholesterol (g/dL)	0.07	-0.05
HDL-Cholesterol (g/dL)	0.22	0.05
Triglyceride (g/dL)	-0.11	-0.04
LDL-Cholesterol (g/dL)	0.02	-0.08
Calcium (mg/dL)	0.08	-0.11
Phosphate (mg/dL)	0.01	-0.17
Intact-PTH (ng/L)	0.02	0.05
FGF23 (pg/mL)	**0.23**[a]	0.10
C-reactive protein (mg/dL)	-0.20	0.06
β2-microglobulin (mg/L)	0.16	**0.35**[b]

Abbreviations: EGF, epidermal growth factor; IL-, interleukin- ; IP-10, Interferon gamma-induced protein 10 (C-X-C motif chemokine ligand 10); MCP-1, Monocyte chemoattractant protein−1; MIP-1α, macrophage inflammatory protein 1α; MIP-1β, Macrophage inflammatory protein-1β; TNFα, tumor necrosis factor α; HDL, high-density lipoprotein; LDL, low-density lipoprotein, calculated based on Friedewald formula, (total cholesterol) - (high-density lipoprotein cholesterol) – (triglycerides/5); intact-PTH, intact parathyroid hormone; FGF23, Fibroblast growth factor 23. Signigficant values are in bold. $^a p<0.05$, $^b p<0.01$

Supplementary Table 3. Bivariate correlations expressed as rho correlations of High density CPP with other variables at baseline in 78 HD patients.

Variables	Rho correlations	
	H-CPP	L-CPP
Age (years)	-0.03	-0.11
Males (%)	0.15	0.07
Dialysis vintage (month)	0.16	**-0.24**[a]
Diabetes mellitus (present)	-0.02	0.15
Cardiovascular disease (present)	**0.23**[a]	0.04
Current Smoking	**0.29**[b]	0.08
Systolic blood pressure (mmHg)	-0.02	0.16
Body mass index (kg/m^2)	0.20	**0.30**[b]
Hemoglobin (g/dL)	0.10	0.10
Albumin (g/dL)	-0.12	**0.23**[a]
Total Cholesterol (g/dL)	-0.20	0.21
HDL-Cholesterol (g/dL)	0.06	-0.09
Triglyceride (g/dL)	-0.14	**0.34**[b]
LDL-Cholesterol (g/dL)	-0.22	0.20
Calcium (mg/dL)	0.11	**0.25**[a]
Phosphate (mg/dL)	**0.39**[c]	**0.27**[a]
Intact-PTH (ng/L)	-0.13	-0.05
FGF23 (pg/mL)	**0.41**[c]	**0.25**[a]
C-reactive protein (mg/dL)	-0.10	0.19
β2-microglobulin (mg/L)	**0.25**[a]	**-0.25**[a]

Abbreviations: EGF, epidermal growth factor; IL-, interleukin- ; IP-10, Interferon gamma-induced protein 10 (C-X-C motif chemokine ligand 10); MCP-1, Monocyte chemoattractant protein−1; MIP-1α, macrophage inflammatory protein 1α; MIP-1β, Macrophage inflammatory protein-1β; TNFα, tumor necrosis factor α; HDL, high-density lipoprotein; LDL, low-density lipoprotein, calculated based on Friedewald formula, (total cholesterol) - (high-density lipoprotein cholesterol) – (triglycerides/5); intact-PTH, intact parathyroid hormone; FGF23, Fibroblast growth fator-23. Signigficant values are in bold. $^a p<0.05$, $^b p<0.01$, $^c p<0.001$

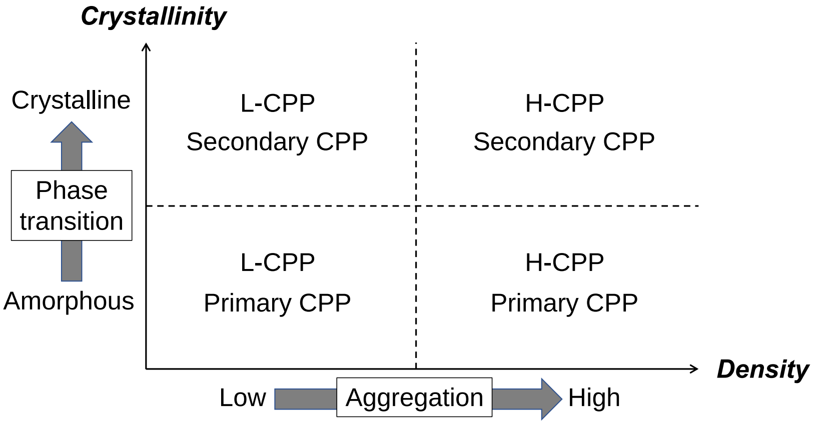

Supplementary Figure 1. **CPPs have been classified by two different axes (crystallinity and density).** Primary CPPs and secondary CPPs are classified based on their crystallinity. primary CPPs are defined as CPPs that do not contain crystalline CaPi, whereas secondary CPPs are defined as CPPs containing crystalline CaPi. L-CPP and H-CPP are classified based on their density. H-CPPs are defined as CPPs that can be precipitated by centrifugation at 16,000g for 120 minutes, whereas L-CPPs are defined as CPPs that cannot be precipitated by centrifugation at 16,000g for 120 minutes. Therefore, four different classes of CPP exist in theory.

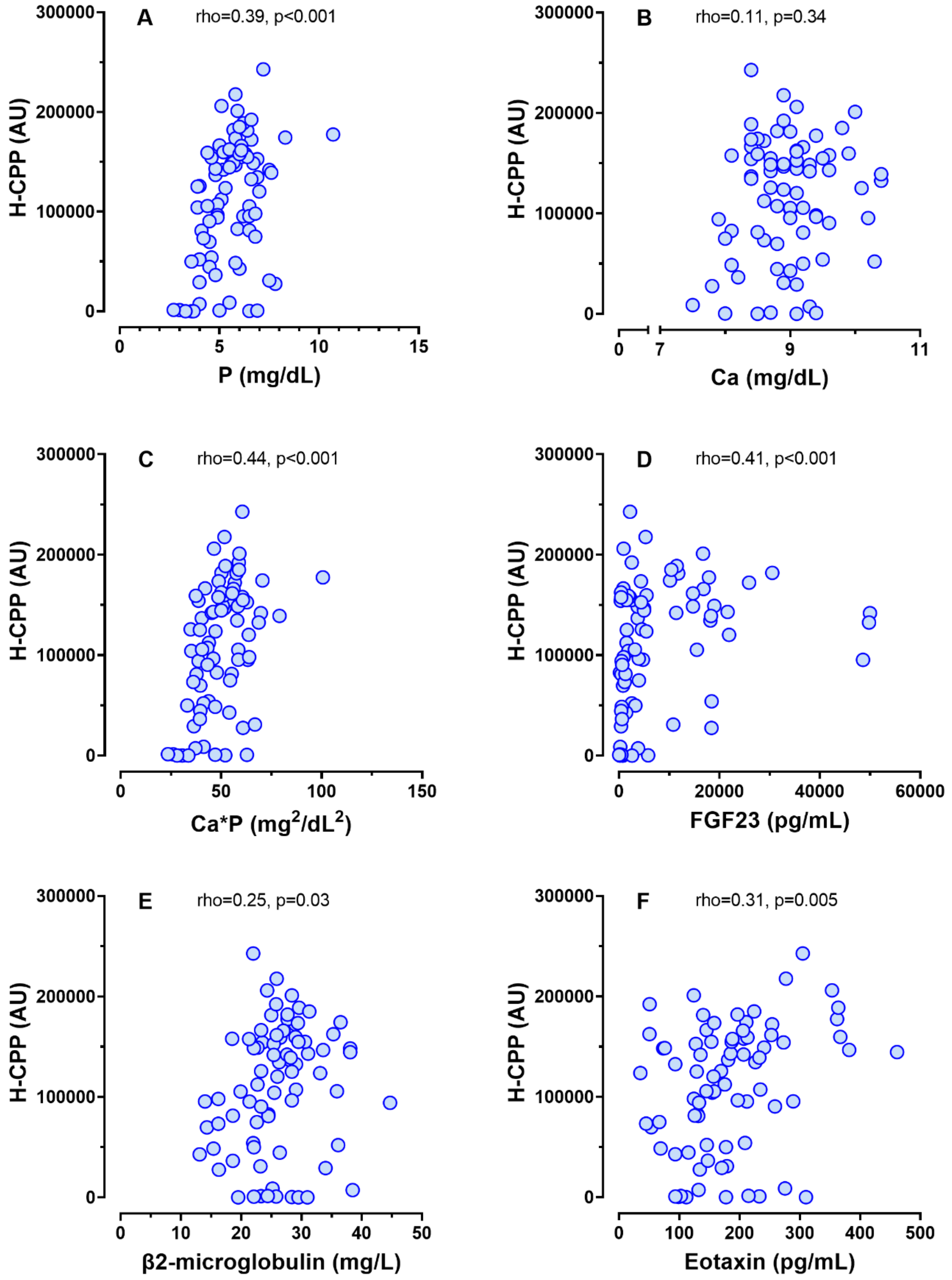

Supplementary Figure 2. Correlations between H-CPP levels and other serum parameters. Correlations of H-CPPs with phosphate (**A**), calcium (**B**), calcium phosphate product (**C**), FGF23 (**D**), β2-microglobulin (**E**) and Eotaxin (**F**). Rho and *p* values were indicated.
